# Awareness survey of so-called Dappou drugs or Kiken drugs (New Psychoactive Substances) among University Students in Japan

**DOI:** 10.1186/s13011-015-0035-0

**Published:** 2015-10-09

**Authors:** Yasuko Fuse-Nagase, Fukumi Saito, Toshie Hirohara, Happei Miyakawa

**Affiliations:** University Health Center, Ibaraki University, 2-1-1 Bunkyo, Mito, Ibaraki 315-8012 Japan; College of Education, Ibaraki University, 2-1-1 Bunkyo, Mito, Ibaraki 315-8012 Japan; Ibaraki University, 2-1-1 Bunkyo, Mito, Ibaraki 315-8012 Japan

**Keywords:** New psychoactive substances, *dppou* drugs, *kiken* drugs, Awareness survey, Japanese university students

## Abstract

**Background:**

Spread of new psychoactive substances (NPS) is a worldwide problem. In Japan, NPSs with psychoactive ingredients are called as “*dappou* drugs” or “*kiken* drugs.” Their potential effect on the Japanese society cannot be ignored.

**Findings:**

We conducted an awareness survey of So-called *Dappou* Drugs or *Kiken* Drugs among the students of Ibaraki University, a national university in Japan, in April 2014. 3976 students (2425 men, 1406 women and 145 unspecified) participated in this study. 2813 (70.7 %) respondents were aware of *dappou* drugs. Only 39.5 % of the respondents selected the option of “ingredients that cause delusions and/or hallucinations may be included” in *dappou* drugs. 23.4 % of the respondents selected “the number of (*dappou* drug) users requiring emergency hospitalization due to acute intoxication is increasing”. Of the respondents, 19 (0.5 %) reported that they had been invited to use *dappou* drugs previously, and 40 (1.0 %) had witnessed and/or heard of somebody close to them using the drugs. Those who drank alcohol every day and those who smoked had a higher chance of witnessing and/or hearing of somebody close to them using *dappou* drugs than those who did not drink or smoke, respectively.

**Conclusions:**

Japanese university students do not have sufficient knowledge about *dappou* drugs or *kiken* drugs to protect themselves from potential drug misuse. It is both important and urgent to educate Japanese university students about the harmful effects of *dappou* drugs; in addition, it is important to provide such knowledge before the students are allowed to legally drink and smoke.

## Findings

### Background

Spread of new psychoactive substances (NPS) is a worldwide problem [1–5]. The United Nations Office on Drug and Crime has reported that NPS are being used in 94 countries worldwide and that 348 NPS had been reported by 2013 [6]. Although the proportion of NPS users in Japan is relatively lower than that in other countries [7–9], the potential effects of NPS on the Japanese society cannot be ignored. In Japan, NPS with psychoactive ingredients are called as “*dappou* drugs” or “*kiken* drugs.” The term “*dappou* drugs” refers to drugs that slip through legal regulations. The term “*kiken* drugs” refers to dangerous or life-threatening drugs. Similar to NPS in other countries, *dappou* drugs or *kiken* drugs are sold under names such as “herbs,” “bath salts,” and “aromatic liquids” but contain some (often more than one) psychoactive ingredients such as synthetic cannabinoids, synthetic cathinones, and/or ketamine derivatives [10, 11]. They are easily available in shops or through the Internet [10, 11]. According to a survey conducted at 2 nightclubs, 21.7 % of those who were joining a club event had ever used *dappou* drugs in the past [12]. *Dappou* drugs or *kiken* drugs are usually sold without any information about their psychoactive ingredients. Thus, users do not know what type of and how much psychoactive ingredients they are consuming. Since 2011, *dappou* drug or *kiken* drug users with medical problems, such as psychosis and disturbance of consciousness have been increasing [13, 14]. A growing number of *dappou* drug or *kiken* drug users have been taken to hospital emergency rooms due to acute intoxication [11, 15], while others have caused traffic accidents or harmed innocent people under the influence of *dappou* drugs or *kiken* drugs [16].

According to a previous awareness surveys, *dappou* drug or *kiken* drug users are younger and have higher education levels than amphetamine/methamphetamine users [14].

We conducted an awareness survey among university students in April 2014 with the aim of determining if Japanese university students have sufficient knowledge and understanding about illegal drugs and *dappou* drugs or *kiken* drugs to protect themselves from the potential negative effects of drug use. In this article, we report the results from a section of the questionnaire that focused mainly on *dappou* drugs or *kiken* drugs.

## Methods

Ibaraki University is a national university located in the metropolitan area of Tokyo, Japan. All students (*N* = 6815) who underwent an annual medical check-up at the university in April 2014 were invited to participate in the survey.

Along with the actual questionnaire, written information was distributed to inform the students about the study. Participation was voluntary and those who agreed to answer the questionnaire participated anonymously. The questionnaire used in a previous survey conducted in April 2009 [17] was revised so that it was suitable to assess the use of *dappou* drugs or *kiken* drugs in addition to illegal drugs. *Kiken* drug is a new term introduced by the government after the survey; therefore, only the term *dappou* drug was used in the questionnaire. The questionnaire also contained questions about each student’s sex, years at the university, smoking habits, and alcohol drinking habits.

Questionnaire items pertaining to *dappou* drugs were extracted from the study and analyzed. Invalid data were excluded. Fisher’s exact test was used to compare two groups. All analyses were performed using SPSS, version 21.

This study was approved by the ethics committee of Ibaraki University.

## Results

### Students’ knowledge and understanding about *dappou* drugs

A total of 3976 students (2425 men, 1406 women, and 145 unspecified) participated in this study. The response rate was 58.3 % (3976/6815), and the completion rate was 86.1 % (3423/3976). Among the respondents, 2813 (70.7 %) were aware of *dappou* drugs. Women were significantly more knowledgeable than men (75.0 % vs. 69.6 %, *p* < 0.01).

Students’ overall knowledge and understanding about *dappou* drugs are presented in Table 1. For the question “What do you think are the differences between so-called *dappou* drugs and illegal drugs? Choose all the options you agree with,” we found that 6.7 % of the students selected the option of “no or little harm to the body and mind,” and 33.8 % selected “slip through legal regulations by altering their ingredients.” Furthermore, 15.5 % of the respondents selected “not illegal to possess or use,” whereas 8.9 % selected “not feeling guilty about possessing or using.” Almost a quarter of the respondents (22.7 %) selected “easily available.” For the question “Choose all the options that reflect your knowledge and understanding about so-called *dappou* drugs,” we found that 39.5 % of the respondents selected “ingredients that cause delusions and/or hallucinations may be included.” While 23.4 % of the respondents selected “the number of users requiring emergency hospitalization due to acute intoxication is increasing,” 42.1 % selected “dangerous and should not be used”; however, there were still 2.0 % of the respondents who selected “would like to use if not illegal.” Significantly more men (3.07 %) than women (1.41 %) responded that they would want to use *dappo*u drugs if they were not illegal (*p* = 0.04).

**Table 1:** Students’ overall knowledge of and understanding about so-called *dappou* drugs

What do you think are the differences between so-called *dappou* drugs and illegal drugs? Choose all the options you agree with	
	Percentage of students who selected the option
No harm or little harm to the body and mind.	6.7 %
Slip through legal regulations by altering their ingredients.	33.8 %
Not illegal to possess or use.	15.5 %
Not feeling guilty about possessing or using.	8.9 %
Easily available.	22.7 %
No idea.	22.7 %
Choose all the options that reflect your knowledge and understanding about so-called *dappou* drugs.	
	Percentage of students who selected the option
Ingredients that cause delusions and/or hallucinations may be included.	39.5 %
Numbers of users requiring emergency hospitalization due to acute intoxication is increasing.	23.4 %
Would like to try to use if not illegal.	2.0 %
Dangerous and should not be used.	42.1 %
No idea.	19.1 %

### Students invited to use or those who had witnessed and/or heard of others using *dappou* drugs

Of the respondents, 19 (0.50 %) reported that they had been invited to use *dappou* drugs previously, and 40 (1.0 %) had witnessed and/or heard of somebody close to them using the drugs.

Those who drank alcohol every day and those who smoked had a higher chance of being invited to use *dappou* drugs than those who did not drink or did not smoke, respectively (3/88 vs. 4/1666, *p* < 0.01 and 6/253 vs. 11/3678, *p* < 0.01, respectively), although the number of respondents being invited to use was relatively small. Similarly, respondents who drank alcohol every day and those who smoked also had a higher chance of witnessing and/or hearing of somebody close to them using *dappou* drugs than those who did not drink or smoke, respectively (5/77 vs. 8/1447, *p* < 0.01 and 10/208 vs. 28/3185, *p* < 0.01, respectively).

## Discussion

### Sampling

To the best of our knowledge, this is one of the first NPS awareness surveys targeting a substantial number of university students in Japan. An important characteristic of this study was the method of data sampling. The sampling method was minimally biased and reflects the real-life situation of Japanese university students. Students were invited to complete the questionnaire at their annual university medical check-up, which approximately 80 % of all university students participate in. Alternatively, if we had recruited students using an Internet solicitation, the participants could have been biased toward students interested in drug use.

### Students’ knowledge and understanding about *dappou* drugs

More than 70 % of respondents were aware of “*dappou* drugs”. However, less than 40 % were aware that *dappou* drugs may contain ingredients that cause delusions and/or hallucinations. In addition, relatively few students were aware that the number of *dappou* drug users requiring emergency hospitalization due to acute intoxication is increasing. Thus, several students were unaware of the harmful effects of *dappou* drugs or *kiken* drugs. Addressing this knowledge gap is critical to students’ safety and wellness.

### Drinking, smoking, and NPS

Students who drank every day and those who smoked had a higher chance of being invited to use *dappou* drugs and witnessing and/or hearing of someone close to them using the drugs. Risky drinking behavior has been associated with substance misuse among college freshmen [18]; however, our results showed that daily habitual drinking made students more vulnerable to *dappou* drug or *kiken* drug use.

### Necessity of education

Although less than 1 % of the students had been invited to use *dappou* drugs, 70.7 % were aware about the drugs. Considering that it is easy to access inappropriate information on the Internet, students have enough opportunities to raise their interest about *dappou* drugs or *kiken* drugs. We believe that students should be educated to have appropriate knowledge to prevent them from using *dappou* drugs or *kiken* drugs.

### Limitations

The response rate was 58.3 % and the completion rate was 86.1 %, which may have biased the results. All study participants were students at a national university, which may have also skewed the results. The responses may have been different to some extent if males and females from a similar age group but a different social background had been surveyed. In a recent report [19], 4 of 144 people (2.8 %) aged 20 to 24 years had seen and/or heard of somebody close to them using *dappou* drugs in the past year, which was higher than the rate observed in our student population.

In this study, we compared responses from students who drank alcohol every day with those from students who did not drink. However, according to the Japanese Ministry of Health, Labor and Wellness, habitual drinking is defined as drinking more than three times a week [20]. The wording of our questionnaire should have followed that definition.

We did not ask the students whether they had used *dappou* drugs. We admit that this question would have been important. However, we believe that some students might have felt upset or offended to be asked a question regarding their own drug use, because the survey was conducted at the time of annual medical check-up and it took place right after freshmen had entered the university. In the Japanese society, it was a reasonable consideration.

If we had targeted specific population but not students in general, the questionnaire could have been expanded to provide more salient information as was in previous surveys targeting mephedrone users in UK [21, 22].

## Conclusions

Japanese university students do not have sufficient knowledge about *dappou* drugs or *kiken* drugs to protect themselves from potential drug misuse. Accurate knowledge is essential for proper protection against *dappou* drug or *kiken* drug use. Thus, it is both important and urgent to educate Japanese university students and other younger students about the harmful effects of *dappou* drugs or *kiken* drugs to prevent them from trying and using the drugs; in addition, it is important to provide such knowledge before the students are allowed to legally drink and smoke.

## References

[1] German CL, Fleckenstein AE, Hanson GR (2014). Bath salts and synthetic cathinones: an emerging designer drug phenomenon. Life Sci.

[2] Helander A, Bäckberg M, Hultén P, Al-Saffar Y, Beck O (2014). Detection of new psychoactive substance use among emergency room patients: results from the Swedish STRIDA project. Forensic Sci Int.

[3] Jerry J, Collins G, Streem D (2012). Synthetic legal intoxicating drugs: the emerging ‘incense’ and ‘bath salt’ phenomenon. Cleve Clin J Med.

[4] Johnson LA, Johnson RL, Portier RB (2013). Current "legal highs". J Emerg Med.

[5] Murphy CM, Dulaney AR, Beuhler MC, Kacinko S (2013). "Bath salts" and "plant food" products: the experience of one regional US poison center. J Med Toxicol.

[6] United nations office on drug and crime. Global overview. In: Global synthetic drugs assessment. Amphetamine type stimulants and new psychoactive substances. https://www.unodc.org/documents/southeastasiaandpacific//2014/05/gsda/clean/2014_Global_Synthetic_Drugs_Assessment_Global_Overview.pdf. 2014. Accessed 1 August 2015.

[7] Burns L, Roxburgh A, Matthews A, Bruno R, Lenton S, Van Buskirk J (2014). The rise of new psychoactive substance use in Australia. Drug Test Anal.

[8] Corazza O, Simonato P, Corkery J, Trincas G, Schifano F (2014). "Legal highs": safe and legal "heavens"? A study on the diffusion, knowledge and risk awareness of novel psychoactive drugs among students in the UK. Riv Psichiatr.

[9] Maxwell JC (2014). Psychoactive substances--some new, some old: a scan of the situation in the U.S. Drug Alcohol Depend.

[10] Kamijo Y, Takai M, Fujita Y, Hirose Y, Iwasaki Y, Ishihara S, Yokoyama T, Yagi K, Sakamoto T (2014). A multicenter retrospective survey of poisoning after consumption of products containing synthetic chemicals in Japan. Intern Med.

[11] Tanibuchi Y, Matsumoto T (2015). [Problems around the “legal highs”(law-evading new psychoactive substances.)]. [Article Japanese] Seishinigaku.

[12] Shimane T, Wada K, HidakaY. Club event raijyousha niokeru ihou drug no ranyo jittai haaku ni kansuru kenkyu. [Research about drug abuse among nightclub event attendants.] [Article in Japanese]http://www.ncnp.go.jp/nimh/yakubutsu/drug-top/data/researchSHIMANE2013_2.pdf. 2013. Accessed 1 August 2015.

[13] Fuse-Nagase Y, Nishikawa T (2013). Prolonged delusional state triggered by repeated ingestion of aromatic liquid in a past 5-methoxy-N. N-diisopropyltryptamine abuser Addict Sci Clin Pract.

[14] Tanibuchi Y, Matsumoto T, Kobayashi O, Wada K (2013). Clinical characteristics of dappou herb use--disorder patients at the drug dependence clinic: a comparison with methamphetamine use-disorder patients.] [Article in Japanese. Seishin Shinkeigaku Zasshi.

[15] Japanese Ministry of Internal Affairs and Communication. Kiken drug ni yoru monoto utagawareru kyukyuhansou no jokyo. [Situation of ambulance transfer under *kiken* drug use.] [Article in Japanese]http://www.fdma.go.jp/neuter/topics/houdou/h26/2609/260919_1houdou/03_houdoushiryou.pdf. 2014. Accessed 1 August 2015.

[16] National Police Agency. Heisei 26 nen no yakubutsu jyuki jyousei. [Situation of drug and fire arm in 2014.] [Article in Japanese]https://www.npa.go.jp/sosikihanzai/yakubutujyuki/yakujyuu/yakujyuu1/h26_yakujyuu_jousei.pdf. 2015. Accessed 1 August 2015.

[17] Nakano T, Takeshita S, Saito F, Miyakawa H (2012). [Awareness survey of illegal drugs such as Marijuana/Hashish among college students.] [Article in Japanese]. Jpn J School Health.

[18] Shimane T, Wada K, Mishima K, Fujiwara M (2009). Association between risky drinking behaviors and risk groups of substance abuse: a study in Japanese college freshmen.] [Article in Japanese. Nihon Arukoru Yakubutsu Igakkai Zasshi.

[19] Wada K, Kyu F, Shimane T. [Awaness Survey of smoking, drinking, and drug.] [Article in Japanese] http://www.ncnp.go.jp/nimh/yakubutsu/drug-top/data/researchJDU2013.pdf. Accessed 11 April 2015.

[20] Japanese Ministry of Health, Labor and Wellness. Habitual drinking. http://www.e-healthnet.mhlw.go.jp/information/dictionary/alcohol/ya-015.html. 2015. Accessed 1 August 2015.

[21] Carhart-Harris RL, King LA, Nutt DJ (2011). A web-based survey on mephedrone. Drug and Alcohol Depend.

[22] Winstock A, Mitcheson L, Ramsey J, Davies S, Puchnarewicz M, Marsden J (2011). Mephedrone: use, subjective effects and health risks. Addiction.

